# Application of EPR Spectroscopy to Examination of Free Radical Formation in Thermally Sterilized Flumetasone

**DOI:** 10.1007/s00723-013-0490-6

**Published:** 2013-10-29

**Authors:** Magdalena Kościelniak-Ziemniak, Barbara Pilawa

**Affiliations:** 1 Department of Biophysics, School of Pharmacy and Laboratory Medicine, Medical University of Silesia in Katowice, Jedności 8, 41-200 Sosnowiec, Poland; 2 Artificial Heart Laboratory, The Zbigniew Religa Foundation of Cardiac Surgery Development, Wolności 345a, 41-800 Zabrze, Poland

## Abstract

Thermal formation of free radicals in flumetasone sterilized according to the pharmaceutical norms at 160, 170 and 180 °C was examined by electron paramagnetic resonance (EPR) spectroscopy. The microbiological analysis was done. Similar free radical concentrations were measured for flumetasone sterilized at 160 and 170 °C. The concentration was considerably higher than that for flumetasone sterilized at 180 °C. Fast spin–lattice relaxation processes, homogeneously broadened EPR lines, and complex free radicals system characterize the heated flumetasone. Free radicals were not observed 30 days after thermal sterilization. Optimal temperatures of sterilization of flumetasone are 160 and 170 °C.

## Introduction

Thermal and radiative sterilization of drugs are used in pharmacy to remove microorganisms from the samples [1–4]. The conditions of sterilization are determined by the norms [1–5]. The aim of the sterilization process is to obtain products free of bacteria [1–5]. The sterility assurance level (SAL) according to the pharmaceutical norms is 10^−6^ [1, 6, 7]. Taking into account the toxic action of free radicals in human organism [8–16], the production of free radicals in the sterilization process is not desired.

In this work, we proposed the application of electron paramagnetic resonance (EPR) spectroscopy at X-band (9.3 GHz) to examine the conditions of thermal sterilization of flumetasone. EPR studies were used together with the standard microbiological tests. The aim of this work was to determine free radical properties and concentrations in this drug sterilized at different temperatures and different times. The best conditions of sterilization of flumetasone were searched for. The temperature and time of thermal sterilization were determined. The optimal conditions of thermal sterilization are those which cause the lowest free radical formation in the drug. The EPR examination of sterilized flumetasone was not performed earlier. The results were compared with those of the EPR studies of free radicals in the other thermally and radiative sterilized drugs [17–26].

## Experimental Procedures

### Samples

**Fig. 1:**
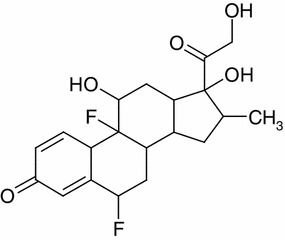
Chemical structure of flumetasone [27]

Flumetasone, a glucocorticosteroid drug, was studied by EPR spectroscopy. Glucocorticosteroids are steroid hormones produced in the adrenal glands. They show a strong antialergic effect [27]. They are used as anti-inflammatories, in conditions such as severe asthma and allergic reactions. Glucocorticosteroids are also used as immunosuppressants to prevent transplant rejection and in certain autoimmune disorders. Flumetasone is anti-inflammatory, synthetic glucocorticosteroid, used topically in aerosol form for the treatment of asthma [27]. The chemical structure of flumetasone is presented in Fig. 1 [27].

### Thermal Sterilization

The examined glucocorticosteroid drug was thermally sterilized according to the Polish Pharmaceutical Norms [1–5]. The following temperatures and times of this process were used: 160 °C during 120 min, 170 °C during 60 min, and 180 °C during 30 min.

**Fig. 2:**
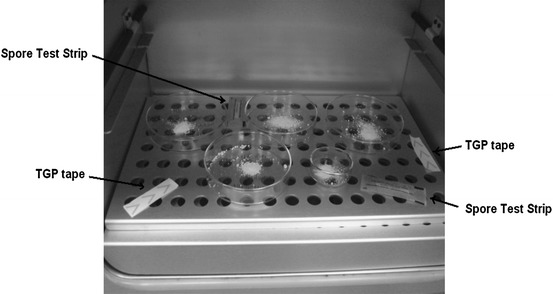
Hot air oven for the sterilization process

Heating processing of the powdered drug samples was performed in a hot air oven with air circulation (Fig. 2). The temperature in the hot air oven was tested using a TGP tape [28]. Fragments of the TGP tape were located in the several positions in the apparatus. They changed color according to the achieved temperature in the environment. The TGP tape location in the hot air oven is shown in Fig. 2.

### Microbiological Analysis

The content of microorganisms in the drug samples were examined by the use of biological tests [29, 30]. The spore test strip with *Bacillus atrophaeus* was placed in the tubes at the temperature of the sterilization process and the color and physical textures were observed. The culture development of these bacteria changed the physical view of the solutions. The bacteria of *Bacillus atrophaeus* which were used during our measurements are the typical strain applied in microbiological analysis.

The spore test strip containing *Bacillus atrophaeus* was located in hot air oven near the sterilized flumetasone during sterilization. The bacterial content in the spore test strip after sterilization was analyzed. For the properly performed sterilization, bacteria should be not found in the spore test strip. The absence of bacteria in the spore test strip indicates the absence of the bacteria in the tested drug, because they were sterilized together.

### EPR Measurements

Paramagnetic centers in the samples were studied using X-band (9.3 GHz) EPR spectroscopy. The first-derivative EPR spectra of the original and the heated samples were measured as the first derivatives. The EPR Radiopan (Poznań, Poland) spectrometer with magnetic modulation of 100 kHz was used. The EPR spectra were collected at room temperature. The total microwave power (*M*_o_) produced by klystron was 70 mW. The spectra were numerically collected by the Rapid Scan Unit and spectroscopic programs of Jagmar Firm (Kraków, Poland). For the numerical acquisition, the LabView program of 8.5 National Instrument Firm was applied. The EPR spectra were recorded 10 min and 10 days after sterilization.

**Fig. 3:**
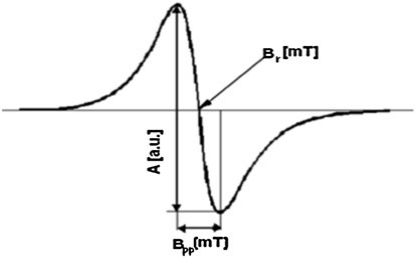
First-derivative EPR spectrum with the parameters: amplitude (*A*), linewidth (Δ*B*_pp_), and resonance magnetic field (*B*_r_)

The values of *g*-factors, amplitudes (*A*), integral intensities (*I*), and linewidths (Δ*B*_pp_) of the EPR spectra of the tested drugs were obtained (Fig. 3). Free radical concentrations (*N*) in the samples were determined.

The EPR measurements were done three times for each of the samples. The maximal values of the errors of the analyzed amplitude (*A*), intensity (*I*), linewidth (Δ*B*pp), *g*-factor, and free radical concentration (*N*) were: ±0.2 (au), ±0.3 (au), ±0.2 (mT), ±0.0002, ±0.2 × 10^18^ (spin/g), respectively (Fig. 3).

The formula of the resonance condition in paramagnetic samples was used to determine *g*-values [31, 32]:

$$ g \, = \, hv \, /\mu_{\text{B}} B_{\text{r}} , $$

where the abbreviations means: *h* Planck constant, *ν* microwave frequency, *μ*_B_ Bohr magneton, *B*_r_ resonance magnetic field.

The microwave frequency (*ν*) was measured on an MCM101 recorder produced by EPRAD Firm (Poznań, Poland). The *B*_r_ values were determined from the EPR lines.

Concentrations of paramagnetic centers (*N*) in the studied samples were compared. The concentration is proportional to the integral intensity (*I*) of the EPR line [31, 32]. The integral intensities were calculated by double integration of first-derivative EPR spectra. Ultramarine was used as the reference for the concentration of free radicals. To obtain the values of concentration, the integral intensities of the spectra of the examined samples were compared with the integral intensity of the ultramarine spectrum. In addition, the second reference, a ruby crystal, was permanently placed in a resonance cavity. For each sample and for the reference ultramarine, the EPR line of the ruby crystal was detected.

The concentration of the paramagnetic centers (*N*) was calculated as follows:

$$ N \, = \, n_{\text{u}} \left[ {\left( {W_{\text{u}} A_{\text{u}} } \right)/I_{\text{u}} } \right]/\left[ {I/\left( {WAm} \right)} \right] , $$

where *n*_u_ the number of paramagnetic center in the ultramarine reference, *W* and *W*_u_ the receiver gains for sample and the ultramarine, *A* and *A*_u_ the amplitudes of ruby signal for the sample and the ultramarine, *I* and *I*_u_ the integral intensities for the sample and ultramarine, *m* the mass of the sample.

Influence of the microwave power (*M*) in the range of 2.2–70 mW on the parameters (*A*, Δ*B*_pp_) of the EPR spectra was analyzed. Spin–lattice relaxation processes in the samples were be characterized by the observation of the microwave saturation of their EPR lines. The power of the microwave saturation of EPR lines increases with the acceleration of spin–lattice relaxation processes [31, 32]. Lineshape parameters of EPR spectra of complex paramagnetic center systems change with the microwave power.

## Results

**Fig. 4:**
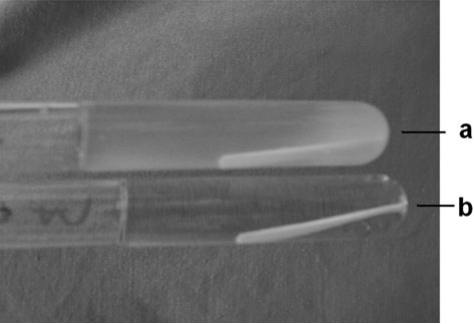
Tryptic soy broth (TSB) with non-sterilized flumetasone (bacteria contents) (**a**) and with sterilized flumetasone (bacteria absence) (**b**)

The microbiological tests brought to light the absence of bacteria in flumetasone sterilized according to the norms [1–4] at 160 °C during 120 min, 170 °C during 60 min, and 180 °C during 30 min. The solutions of the tryptic soy broth (TSB) with the sterilized drug were clear. The photos of the TSB with the addition of non-sterilized flumetasone with the bacterial content and the TSB with the sterilized drug with the absence of bacteria are compared in Fig. 4a, b, respectively.

**Fig. 5:**
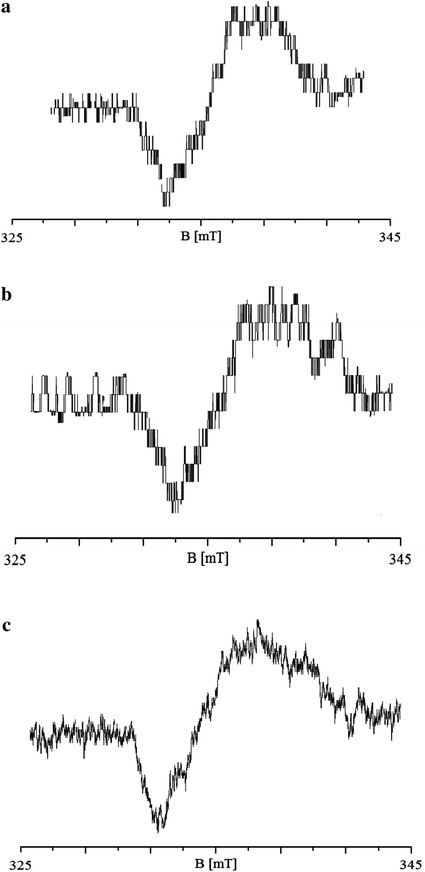
EPR spectra of flumetasone sterilized at temperatures 160 °C (**a**), 170 °C (**b**), and 180 °C (**c**). The spectra were measured at room temperature with the very low microwave power of 2.2 mW to avoid the microwave saturation effect

**Fig. 6:**
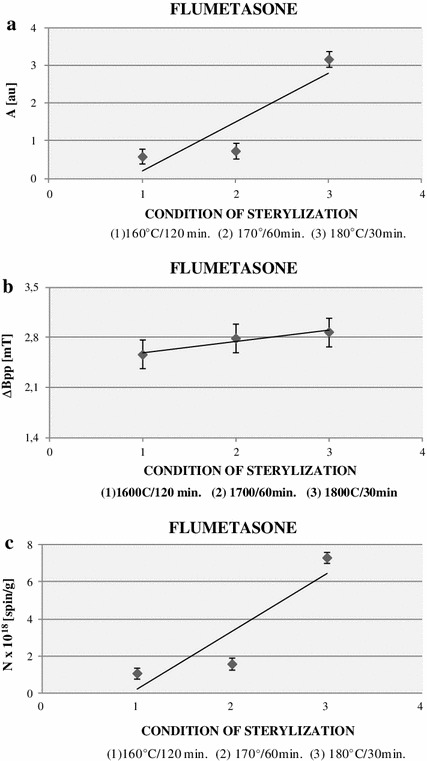
Comparison of amplitudes (*A*) (**a**) and linewidths (Δ*B*_pp_) (**b**) of EPR spectra, and free radical concentrations (*N*) (**c**) in flumetasone sterilized at temperatures 160, 170, and 180 °C. The spectra were measured with the microwave power of 2.2 mW at room temperature

EPR spectra were not detected in the original flumetasone sample. EPR spectra were measured in flumetasone heated at all tested conditions of thermal sterilization. The spectra of flumetasone sterilized at 160 °C (120 min), 170 °C (60 min), and 180 °C (30 min) were the broad lines with linewidths Δ*B*_pp_ of 2.56–2.88 mT. The EPR spectra are shown in Fig. 5. The parameters of these spectra and free radical concentrations (*N*) in the drug are presented in Table 1 and in Fig. 6. The following parameters: *g*-values, amplitudes (*A*), and linewidths (Δ*B*_pp_) of the EPR spectra of the tested drug sterilized at different temperatures and during the respective times, are compared in Table 1. As one can see, free radicals were formed during sterilization of the studied glucocorticosteroid drug. The spectroscopic examination indicated that free radicals were quenched in flumetasone after 30 days from thermal sterilization. After storage of heated flumetasone during 30 days, the EPR spectra were not detected.

**Table 1:** Free radical concentration (*N*) in the sterilized drug samples, and the EPR spectra parameters: *g*-factor, and linewidth (Δ*B*_pp_)

Glucocorticosteroid drugs	Conditions of sterilization		*N* × 10^18^ (± 0.2 × 10^18^ spin/g)	*g* (± 0.0002)	Δ*B*_pp_ (± 0.02 mT)
	*T* (^o^C)	*t* (min)			
	160	120	1.1	1.9946	2.56
Flumetasone	170	60	1.6	1.9947	2.79
	180	30	7.3	1.9977	2.88

The data for the measurement with microwave of 2.2 mW at room temperature

The amplitude (*A*) of EPR lines of flumetasone increases with the increase in temperature of sterilization from 170 to 180 °C (Fig. 5a). Similar amplitudes (*A*) were obtained for the EPR spectra of flumetasone sterilized at 160 and 170 °C (Fig. 5a). The linewidth (Δ*B*_pp_) of EPR spectrum of flumetasone increased with the increase in the temperature of sterilization (Fig. 5b). The difference between free radical concentrations (*N*) of flumetasone sterilized at 160 and 170 °C was not high (Fig. 5c). The strong increase in the free radical concentration (*N*) value was observed for flumetasone sterilized at the highest temperature (180 °C) (Fig. 5c).

The *g*-values for free radicals in flumetasone were in the range of 1.9946–1.9977 (Table 1).

**Fig. 7:**
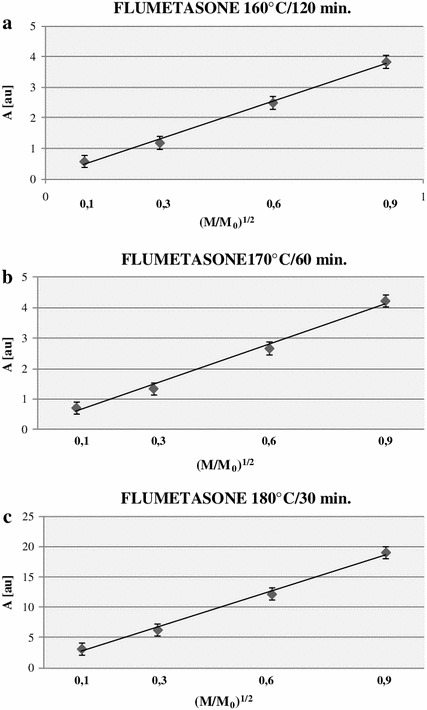
Influence of the microwave power (*M*) on the amplitude (*A*) of EPR spectra of flumetasone sterilized at 160 °C (**a**), 170 °C (**b**), and 180 °C (**c**). *M*_o_ (70 mW) is the total microwave power produced by the klystron. The spectra were measured at room temperature

**Fig. 8:**
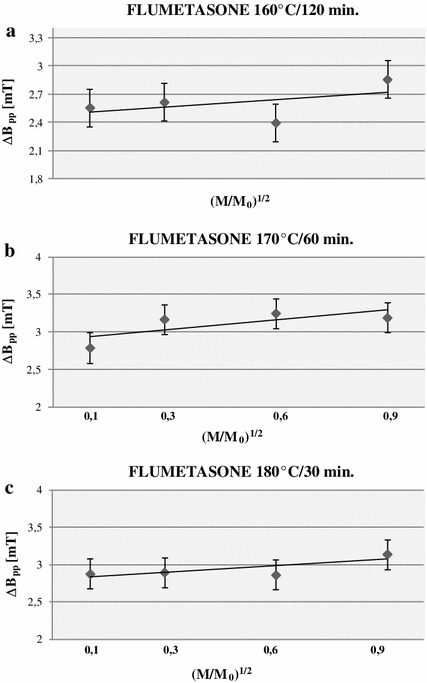
Influence of the microwave power (*M*) on the linewidth (Δ*B*_pp_) of EPR spectra of flumetasone sterilized at 160 °C (**a**), 170 °C (**b**), and 180 °C (**c**). *M*_o_ (70 mW) is the total microwave power produced by the klystron. The spectra were measured at room temperature

The lineshape of the EPR spectra of all tested sterilized flumetasone samples indicated the asymmetry of the spectra (Fig. 5). The asymmetry changed with the increase in the microwave power. The amplitudes and linewidths of these spectra depended on the microwave power. The influence of the microwave power on amplitudes (*A*) and linewidths (Δ*B*_pp_) of the EPR spectra of sterilized flumetasone is shown in Figs. 7 and 8, respectively. Amplitudes of all recorded EPR spectra increase with the increase in the microwave power (Fig. 7). The low increase in linewidths (Δ*B*_pp_) of the EPR spectra was observed (Fig. 8).

## Discussion

EPR studies show the diamagnetic character of the original sample of flumetasone. This sample located in the magnetic field did not absorb microwaves and the EPR spectra were not obtained even at the high microwave power (70 mW), at the high receiver gain of the signal, and after 200 times accumulation of the lines. The paramagnetic impurities do not exist in the original drug, so the chemical structures were not disruptive, and the eventual defects did not affect the results. EPR tests of the purity of the drug samples were proposed in our earlier works [17–23, 25, 26]. The practical and economical importance of such studies results from the low costs of these experiments, and from the non-destructive character of them. The former experiments may be planned for same spectroscopically tested samples.

Only the microbiological tests are usually used to check the effect of sterilization as the process which removes microorganisms from the drug. The hard conditions of thermal sterilization such as the high temperatures may produce free radicals in the treated drugs, so this method may not be used for all pharmaceutical samples. The sterilized drug should not contain free radicals, because they may decrease its effectiveness in organism. Free radicals of drug may also interact with tissues and damage their structures. EPR spectroscopy may be used as an additional method for microbiological tests to obtain the answer to the question about the applicability of the sterilization conditions for the individual sample. Such EPR analyses were performed, for example, for antibiotics [22, 25] and the other glucocorticosteroid drugs [17–21, 23]. In this work, EPR was applied to examine the use of thermal sterilization for flumetasone.

After sterilization, independent of the temperatures, flumetasone was paramagnetic. Sterilized flumetasone revealed EPR signals (Fig. 5). The free radical concentrations in the samples were about 10^18^ spin/g (Table 1; Fig. 6c). The results indicated that flumetasone may be thermally sterilized at temperatures 160 °C (120 min) or at 170 °C (60 min). These conditions of sterilization process produced the lowest amount of free radicals in flumetasone (Table 1; Fig. 6c). In addition, these free radicals differentiate after storage of this drug. Free radicals were not detected also in flumetasone sterilized at 180 °C (30 min) after 30 days, but this process produced the highest amount of free radicals in the tested drug (Table 1), so we did not propose such conditions for killing bacteria in flumetasone. It is expected that the chemical structure of flumetasone heated at 180 °C was damaged. The effect of ‘quenching’ of free radicals in the sterilized flumetasone during storage was probably caused by interactions with oxygen. A similar effect was observed for the other drugs, for example, for thermally and radiative sterilized aminoglycoside antibiotics [22, 25].

The asymmetrical shape of the spectra of sterilized flumetasone and its changes with the increase in the microwave power indicated that different types of free radicals exist in the heated drug (Fig. 5). The asymmetrical spectra were the result of the superposition of several components. The resultant EPR spectra are the sum of these lines, so they are not symmetrical. The EPR lines of the individual type of free radicals changes differently with the microwave power, and the resultant shape of the EPR spectra was changed. The chemical structure (Fig. 1) of flumetasone indicated that different chemical bonds may be ruptured during heating, and the major localization of the unpaired electrons in the samples is expected. Similar effects were obtained for thermally and additive sterilized ampicillin, piperacillin and penicillin [22, 25].

EPR lines of thermally sterilized flumetasone were not saturated up to the microwave power of 70 mW (Fig. 7). The fast spin–lattice relaxation processes existed in the samples. This effect is based on the theory of spin–lattice relaxation processes in paramagnetic samples [31, 32].

The increase in the linewidth of the EPR spectra of thermally sterilized flumetasone (Fig. 8) and the complex shape of EPR spectra indicated homogeneous and inhomogeneous broadening of the EPR lines. The EPR lines of other drugs sterilized by gamma irradiation were homogeneously broadened [25].

Summing up, mainly two methods of sterilization are used for drugs [1–7, 18]: thermal sterilization for drugs, which are not destroyed at high temperatures and radiative sterilization by gamma irradiation for other drugs. These two types of sterilization kill microorganisms, but sterilization without the damage of the chemical structure of drug should be used. Thermal sterilization is better from the economic point of view, so we tested different drugs after this process [17–26]. If the high amount of free radicals appears during thermal sterilization, this method is rejected, because the chemical structure of the drug is not stable at the high temperature. Then the radiative sterilization is proposed as the second possible method.

An individual drug should be sterilized, and there are two methods to be used, so we search for the method which does not produce free radicals or which produces less free radicals in this drug. In any case, the drug should be sterilized. EPR spectroscopy let us demonstrate the method of sterilization which produces less effect on its structure, and this method should be applied in practice. For flumetasone, thermal sterilization at 180 °C was rejected, because of the high concentration of free radicals. Thermal sterilization at 160 and 170 °C produces the lower amounts of free radicals, so these temperatures should be used in practice. We propose to introduce EPR spectroscopy to process the production of drugs.

The performed examination indicated that EPR spectroscopy is a useful method to obtain the optimal conditions of thermal sterilization of flumetasone. The results are an example of the application of EPR spectroscopy in pharmacy. Microbiological and spectroscopic analyses are done to examine the individual drugs before their application in organism.

## Conclusions

EPR studies of flumetasone indicated the following:

1. Paramagnetic impurities do not exist in the non-heated flumetasone as shown by the absence of EPR signals.
2. Free radicals were formed in flumetasone sterilized according to the pharmaceutical norms at 160 °C (120 min), 170 °C (60 min), and 180 °C (30 min), EPR spectra were measured for all the samples.
3. Complex character dependent on microwave power EPR spectra of thermally sterilized flumetasone indicated that different types of free radicals exist in these heated drugs.
4. The influence of the microwave power on amplitudes of the EPR spectra of thermally sterilized flumetasone indicated fast spin–lattice relaxation processes in this drug. The spectra were not saturated up to the microwave power of 70 mW.
5. The mixture of homogeneous and inhomogeneous broadening of the EPR spectra of thermally sterilized flumetasone was obtained.
6. Taking into account the lowest free radicals concentrations, the optimal sterilization of flumetasone is the process at 160 °C (120 min) and at 170 °C (60 min).
7. Free radicals formed during thermal sterilization in flumetasone were not stable; they were not detected after 30 days of storage.
8. Both microbiological and EPR spectroscopic studies are the useful methods to examine the optimal conditions of thermal sterilization of flumetasone.
